# Study protocol for PIANo-1: Personalized Investigation of music’s effect on Attention in a series of N-of-1 trials

**DOI:** 10.1016/j.conctc.2026.101606

**Published:** 2026-01-22

**Authors:** Thomas Gärtner, Fabian Stolp, Bert Arnrich, Stefan Konigorski

**Affiliations:** a Digital Health Cluster, Hasso Plattner Institute for Digital Engineering, University of Potsdam, Potsdam, Germany; b Hasso Plattner Institute for Digital Health at Mount Sinai, Icahn School of Medicine at Mount Sinai, NY, USA

**Keywords:** N-of-1 trial, Study protocol, Attention, Sensor data

## Abstract

**Background:**

Focus and concentration are influenced by various environmental factors, such as listening to music. Recent research highlights the individualized nature of music’s effects on concentration, as responses vary significantly between individuals based on music genres and personal preference. Traditional population-based studies often obscure these between-person differences, while N-of-1 trials, which are individual crossover trials, can provide a personalized approach by allowing each participant to serve as their own control. This study design is particularly suited for examining how self-selected music genres might enhance or alter concentration in each individual. By leveraging an N-of-1 trial design, this study aims to contribute to the growing body of research investigating personalized cognitive interventions, providing insights into individual effects and variations in response to music.

**Methods:**

The study will include approximately 23 participants, who will be allocated to a block-randomized sequence with two cycles, each consisting of 3-min periods of listening to music (intervention, A) and 3 min of no music (control, B) in random order. Study participants will select one of fourteen predefined music genres, with or without lyrics, as their intervention. A playlist with preselected songs from this genre will be played and compared to not listening to music. To minimize the effects of carryover and concentration loss during the study, a 1-min break is planned between each period, resulting in a total duration of around 15 min. Concentration will be assessed by the number of correct classifications of a digital version of the Stroop test within each period. After each period, a short questionnaire will be administered to collect self-assessed concentration and stress scores. Additionally, physiological biomarkers will be assessed using wearables such as Electroencephalography, Heart Rate Variability, Electrodermal Activity, and eye- and pupil-movement data. In the statistical analysis, Bayesian generalized linear mixed models will be used to estimate the intervention effects of music on correct answers of the Stroop task on the individual level and population level.

**Discussion:**

This study will provide insights into the personalized effects of music on concentration, providing a blueprint for individuals on how they may test and improve their concentration.

## 1 Introduction

The impact of music on cognitive performance, particularly on concentration and attention, has been a focal point of research in psychology and neuroscience. In 1976, Parente investigated the performance of the Stroop task in conditions (1) listening to preferred music, (2) least preferred music, and (3) control (no music), resulting in better performances of no music and preferred music compared to least preferred music [1]. These results were not fully aligned with a previous study, where noise was reducing the time for the Stroop task [2], when assuming music is defined as a form of noise [3].

The so-called *Mozart effect*, a phenomenon suggesting that listening to Mozart’s compositions can improve spatial task performance, was first investigated in 1993 by Rauscher et al. [4]. In this study, the performance of three sets of standard IQ spatial reasoning tasks was compared among 36 study participants under 3 conditions: (1) listening to Mozart’s *Sonata for two pianos in D major, K488*; (2) listening to a relaxation tape; and (3) silence. No personal preferences were considered in this study. However, subsequent studies, including those by Steele et al. have failed to replicate the results, calling into question the robustness of the Mozart effect and suggesting that the influence of music on concentration may be more nuanced than previously thought [5]. Other studies have investigated the relationship between arousal and mood and their impact on performance in spatial tasks, while the Mozart effect was considered an artifact of this relationship [6], [7].

Following that, a range of studies have examined how people react differently to music [8], [9], [10]. In 1997, Furnham and Bradley [8] compared the effects of being exposed to background pop music or silence in introverts and extroverts on different tasks, such as memory tasks and reading comprehension. They showed that introverts tend to perform worse under music conditions, especially in a memory task after 6 min. In 2002, Furnham and Strbac [9] compared silence with predefined unreleased Garage-style songs and noise. They found a better overall performance under silence compared to music and noise. In a reading comprehension task, they identified a performance difference between introverts and extroverts in the music and noise conditions. In contrast, in a prose recall task and a mental arithmetic task, they did not identify a difference [9]. Cassidy et al. compared the effect of listening to high arousal music, low arousal music, noise, and silence in introverted and extroverted individuals. Their results indicated that introverts were performing better on immediate recall, free recall, and delayed recall tasks, while there were no differences with respect to a Stroop task [11]. Besides studies of personality, Wu and Shih showed that background music could improve the attention performance of musicians more compared to non-musicians [10]. In 2025, Clark and Leal investigated the effect of prominent music on learning behavior. Based on their study, they suggested that music interventions do not uniformly impact memory and need to be personalized [12].

For investigation of the association between personality traits and music genres, using a classification model for music genres can be helpful. Rentfrow proposed a five-factor model called MUSIC, how music can be classified into (1) a mellow factor (smooth and relaxing), (2) an unpretentious factor (sincere and rootsy), (3) a sophisticated factor (classical, operatic, world, jazz), (4) an intense factor (loud, forceful, energetic), and (5) a contemporary factor (rhythmic, percussive) [13]. Greenberg associated those music factors with personality using the empathizing-systemizing theory [14]. Similar studies have used music classification to associate listening behaviors and emotions [15], [16], [17].

The specific characteristics of music that induce relaxation have been examined in experimental contexts. For example, research by Shepherd et al. compared silence, aircraft noise, and different music genres, including monaural beats at specific frequencies, to a composition called *Weightless*, purportedly designed to be ”the most relaxing song in the world” [18]. Their findings suggest that structured, low-tempo music lowering arousal levels may be more effective compared to silence or certain environmental noises [18]. Lui and Grunberg employed skin conductance to assess how music versus silence affects arousal, concluding that silence can be as influential as music in managing arousal levels during stressful interactions [19]. Shih et al. investigated the effect of background music without lyrics on attention performance measured through Chu’s attention test within a randomized controlled trial [20]. In another study, Huang and Shih showed that background music influences workers’ attention: performance was dependent on the listener’s fondness rather than the type of music [21]. Souza and Barbosa investigated whether there is an effect-difference between music with and without lyrics. In their study, music with lyrics hindered verbal memory, visual memory, and reading comprehension; music without lyrics, such as hip-hop or Lo-Fi, did not show a significant effect [22].

In the medical context, music therapy has gained more popularity over the last decade, such as for anxiety and pain [23]. A variety of studies have explored the nuanced effects of music on stress and anxiety, with findings suggesting that different genres and tempos yield varying outcomes [24], [25], [26]. For instance, Labbé et al. examined different types of music and their effects on stress, anxiety, and anger, finding that particular types of music can serve as effective coping mechanisms for stress reduction depending on the listener’s pre-listening mental state [24]. Further supporting this, a randomized controlled trial by Lee et al. found that music therapy significantly influenced the cardiovascular and autonomic nervous systems in stress-induced university students, highlighting physiological changes that contribute to stress reduction and enhanced well-being [25].

From a neurophysiological perspective, music has been shown to modulate brain activity associated with attention, cognitive control, and emotional processing. Electroencephalography (EEG) studies indicated that listening to music can alter cortical oscillatory patterns, such as frontal theta and alpha activity, which have been linked to attentional engagement and cognitive workload [27], [28], [29]. Furthermore, music has been reported to influence autonomic nervous system activity, reflected in physiological measures such as Heart Rate Variability (HRV) and Electrodermal Activity (EDA), which are sensitive to changes in arousal, stress, and task engagement [30], [31]. These research studies have varied from investigating the overall effect of listening to a specified music genre on concentration, the individual response based on personal characteristics or music preferences, to the effect on different emotions based on the music characteristics.

In our study, we aim to investigate the effect of music from a self-selected music genre on individuals’ concentration, particularly when focusing on attention-based tasks. By collecting baseline data about personality, music preferences, and listening fondness as well as physiological sensor data, we will further explore diverse interactions of music.

### 1.1 Objectives

This study investigates the effect of music on concentration through a rigorous experimental design, assessing individual changes in attention levels. By including various music genres chosen by the study participants, this study will contribute to understanding the role of music in cognitive performance, ultimately providing insights into whether and potentially how music can enhance concentration.

The study addresses one primary and two secondary research questions.

• *Primary research question:* Does listening to music from a self-selected genre influence cognitive task performance compared with a no-music condition, on average on the population level and for how many individual participants?

Secondary research questions:

1. To what extent can physiological sensor data (e.g., Electroencephalography (EEG), Heart Rate Variability (HRV), Electrodermal Activity (EDA), Eye Tracking) serve as proxies for concentration changes, as measured by the cognitive task?
2. To what extent do between-person differences in intervention effects on cognitive task performance be explained by effect modifiers (e.g., music preference, baseline characteristics, physiological traits)?

### 1.2 Trial design

We will conduct a series of personalized N-of-1 trials comparing intervention (**A**, listen to music) and control (**B**, do not listen to music). Each study participant will select a music genre, which they believe will most positively affect their concentration. Each trial consists of two cycles, each containing two treatment periods in a block-randomized sequence of intervention period A and control period B. This yields four possible treatment sequences, *ABAB*, *ABBA*, *BAAB*, and *BABA*. Each treatment period lasts for 3 min, followed by a questionnaire and a 1-min pause. During the treatment periods, the participants perform a digital adaptation of the Stroop test.

Fig. 1Results of Sample Size Calculation.Fig. 1

**(a):**
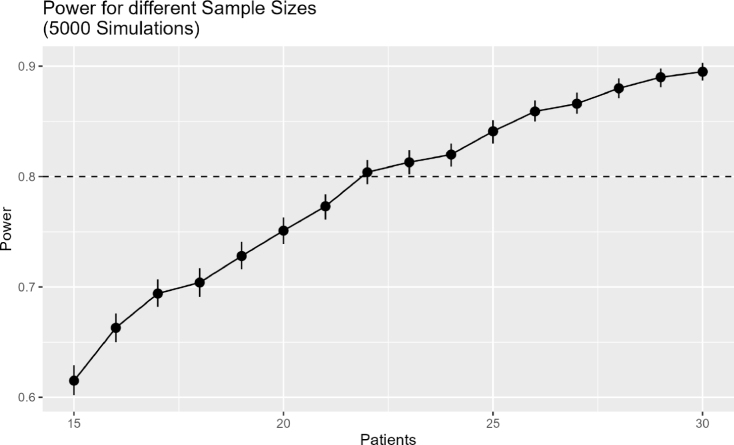
Sample size calculation with fixed $\sigma^{2}=0.05$.

**(b):**
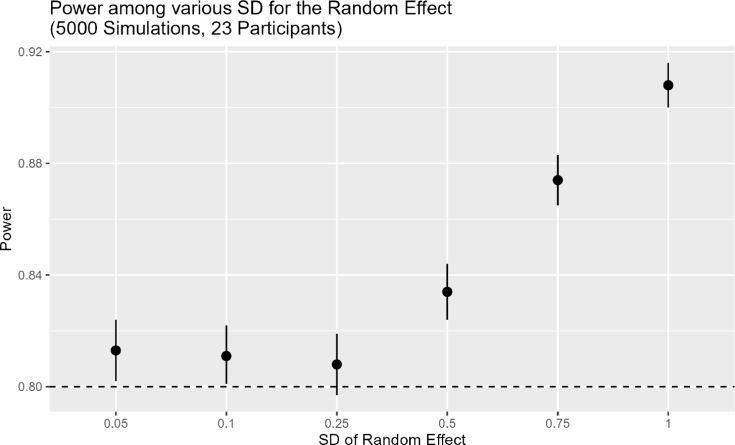
Confirm Power for different values of $\sigma^{2}$.

## 2 Methods

### 2.1 Participants

#### Study setting

The study will be conducted at the Hasso Plattner Institute in Potsdam, Germany. A designated room will be prepared with necessary equipment, including a 2.1 speaker system, screen, mouse, and keyboard, to facilitate the study procedures. Participants will be provided with sensors that are connected to a computer system for data collection. Before the study start, the room will be adequately ventilated to ensure a suitable environment. The participant will be alone in the room during the intervention and control phases.

#### Eligibility criteria

The study population focuses on adults with a minimum age of 18 years. Inclusion and exclusion criteria are specified in Table 1.

**Table 1:** Inclusion and exclusion criteria for the study.

Inclusion criteria	Exclusion criteria
– Fluent in German or English	– Loss of hearing
– Age >=18	– Presence of colorblindness
– Ability to read	– Presence of a neurological disease
– Informed consent	– Presence of epilepsy
	– Presence of a neurodevelopmental disorder
	– Substance abuse (for example, alcohol, drugs, or other)
	– Doctor’s recommendation (or self-assessment) not to perform stressful tasks
	– Participation in another intervention study during the study period

Participants will be asked to give electronic consent, and a physical copy of the consent form will be handed to the participants. Patients or members of the public were not involved in the design, conduct, reporting, or dissemination plans of this research.

#### Sample size

We estimated the required sample size (i.e., number of participants) for testing a population-level average difference in the primary outcome (Stroop test, see Section 2.3) between periods of intervention condition (self-selected music) and control condition (no-music) through simulation studies. The simulation was performed in R using lme4, with seed 123 for reproducibility. We assumed a correct answer rate of 95% in the no-music condition and 96% across all self-selected music conditions based on pilot testing of the digital version of the Stroop task. That is, we assumed a difference in correct answer rate between the conditions of 1%. We assumed 150 observations per 180-second period based on an average response rate of 1.2 s per answer. This yielded about 600 observations per participant across all four periods. Corresponding to those values, we simulated data of our binary outcome variable $Y_{ij}$ (0 = incorrect response, 1 = correct response) for patient i at time point j (subsequently numbered throughout all periods) with a generalized linear mixed model (GLMM) using an intercept of $\beta_{0}$, a fixed effect $\beta_{1}$ of treatment $A_{ij}$ (0 = control condition, 1 = intervention) and we assumed a random effect $u_{i}∼N\left(0,\sigma_{u}^{2}=0.05\right)$ for each individual:

(1) $$\beta_{0}=\mathrm{logit}\left(0.95\right)$$

$$\beta_{1}=\mathrm{logit}\left(0.96\right)-\mathrm{logit}\left(0.95\right)$$

$$p_{ij}={\mathrm{logit}}^{-1}\left(\beta_{0}+\beta_{1}\cdot A_{ij}+u_{i}\right)$$

$$y_{ij}=\text{Bernoulli}\left(p_{ij}\right)$$

We simulated 5000 series of N-of-1 trials for each of the following number n of participants: n∈[15,16,…,29,30]. We used a fixed treatment effect $\beta_{1}$, random effect $u_{i}$, and the outcome was randomly drawn from a Bernoulli distribution as shown in Eq. (1). The seed was set to ‘123’.

Next, for each n, we analyzed each of the 5000 series of N-of-1 trials using the generalized linear mixed model (GLMM) defined in Section 2.5. For each n, we estimated the power as the ratio of p-values of the Wald z-test for $\beta_{1}$ that were <0.05 across the 5000 series of trials and calculated the 95% confidence interval for the estimated power based on the Monte Carlo error of the estimated power:

(2) $${\text{CI}}_{\text{upper}/\text{lower}}=\text{Power}\pm 1.96\cdot \sqrt{\frac{\text{Power}\cdot \left(1-\text{Power}\right)}{5000}}$$

Fig. 1(a) shows that the estimated power was 80.4% (95% CI: 79.3%–81.5%) with 22 patients and 81.3% (95% CI: 80.2%–82.4%) with 23 patients. In a second step, we iterated over different values of $\sigma_{U}^{2}\in \left\{0.1,0.25,0.5,0.75,1\right\}$ and calculated the corresponding power and its confidence interval. Fig. 1(b) shows that all estimated powers are above the required power of 80%, while only with a standard deviation of $\sigma_{u}^{2}=0.25$ the confidence interval of the power includes 80% (79.7%–81.9%). Hence, we aim for 23 participants in our trial.

On the individual level, assuming 600 available data points, this would allow for the estimation of a treatment effect in the improvement of correct answers from 95% to 96% with a 95% confidence interval width of $2\cdot 1.96\cdot SE\left(\overset{ˆ}{\beta_{1}}\right)=0.117$.

#### Recruitment

The study population will be recruited from the University of Potsdam and the surrounding community until reaching 23 participants. The study will be advertised on several channels: posters at the university, announcements on the institutional website, email distribution lists, and social media channels (LinkedIn, Instagram). The material will include the title of the study, a short introduction, a contact address, and a reference to the detailed project website. Interested individuals can contact the study team by email promoted in the material. Upon contact, potential participants will receive further information about the study and a short eligibility screening. Eligible individuals will be invited to schedule an on-site appointment. At the beginning of the session, the study purpose and procedures will be explained, and written informed consent will be obtained before participation. A thank you gift in the form of sweats will be offered after finishing the trial, but no course credits nor financial incentives will be given to the study participants.

### 2.2 Intervention

#### Description

Within the study, we investigate the effect of listening to music from a self-selected genre compared to no-music condition. At the start of the study, we ask participants to choose the music genre that they anticipate will have the greatest positive impact on their concentration from a preselected list. Participants can choose from fourteen different prespecified genres shown in Table 2, where in three of the genres, the participant can specify whether the lyrics should be in German or English, resulting in a total of 17 different sets of songs. The genres are selected based on their popularity and usage in other studies. With the selection of genres, we tried to cover all five factors of the MUSIC model to a certain extent. By providing German and English lyrics to Alternative-Indie, Rock, and Pop music, we allow the participant to select their own preferences, as lyrics contain speech, which might interfere with the task. As the study is conducted in Germany, it is expected that a subset of participants will prefer German lyrics, while other participants will prefer English lyrics.

For each chosen genre, two well-known songs have been selected by the study team. The songs are chosen based on their popularity within the genre and length (minimum 3 min), and only studio recordings will be selected. Data on attributes of the songs, such as key, pitch, or tempo, are gathered through the open platform *Chosic*. The selected songs will not be disclosed to participants before the trial. For selecting the genre, example songs will be played to the individual so that the participant can select the genre properly. A list of all songs used for intervention can be found in Supplementary Table 1.

**Table 2:** Music genres grouped based on the MUSIC factors [13].

Factor	Music genre
Mellow	Ambient
	LoFo-Jazz
	Alternative-Indie^a^, ^b^

Unpretentious	Folk

Sophisticated	Cinematic
	Classic

Intense	Classic-Rock
	Metal
	Rock^b^
	Alternative-Indie^a^, ^b^

Contemporary	EDM
	Hip-Hip
	K-Pop
	Pop^b^
	Techno

a Alternative-Indie could be considered as Mellow and Intense.

b The participant can select English or German lyrics.

During the intervention phases, the songs will be presented in a randomized order. For that, the sound will be played on an external 2.1 speaker system in the room. Study participants control the volume on their own during the study. However, the study team will monitor that the volume does not exceed 80 dB and will be at a minimum of 50 dB.

In the baseline phase, no music and no noise will be played to the study participants.

#### Allocation and blinding

The phases within the study will be randomized within each block (*AB* or *BA*) to minimize the risk of bias when combining the results from the individual trials [32]. The randomization will be independent of previous intervention allocations, and the package *numpy* from *python* will be used.

Due to the nature of the intervention, neither the participants nor the investigators conducting the sessions can be blinded to the assigned condition. The outcome measures are objective and automated, which reduces the potential for assessment bias.

### 2.3 Measurements, effect modifiers and additional variables

#### Outcomes

The primary outcome in our study is concentration, which will be assessed through the number of correct responses from the participant on a concentration task. For that, we will use an adapted version of the Stroop test tailored for a digital environment. The Stroop test is a well-established neuropsychological assessment designed to evaluate the ability to inhibit cognitive interference and maintain selective attention [33], [34]. In our adapted Stroop test, study participants will view words displayed in different colors (see Fig. 2). When the ink color matches the word’s meaning, participants are instructed to press the key *’r’*, and the key *’w’* if they do not. The system will further record the reaction time (i.e., the time between when the word is shown until a key is pressed). This modification ensures the test is compatible with the digital setup while maintaining its ability to measure concentration and cognitive control.

For answering the secondary research questions, sensor data defined below will be used.Fig. 2Screenshots of the adapted Stroop test. The top-left corner shows the phase’s remaining time, and a gray progress bar below the word indicates the time left for the current word.Fig. 2

**(a):**
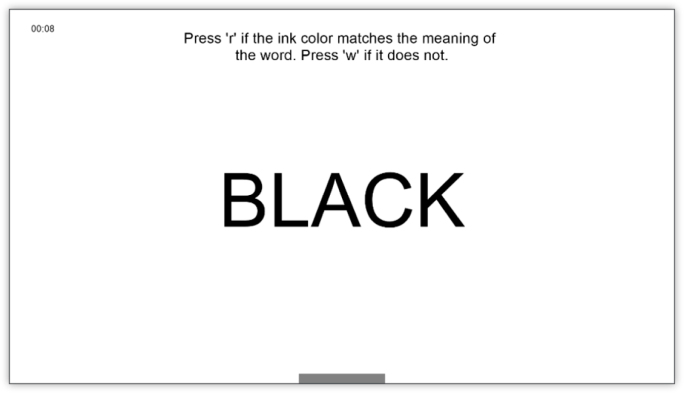
Example, where the ink color matches the word meaning.

**(b):**
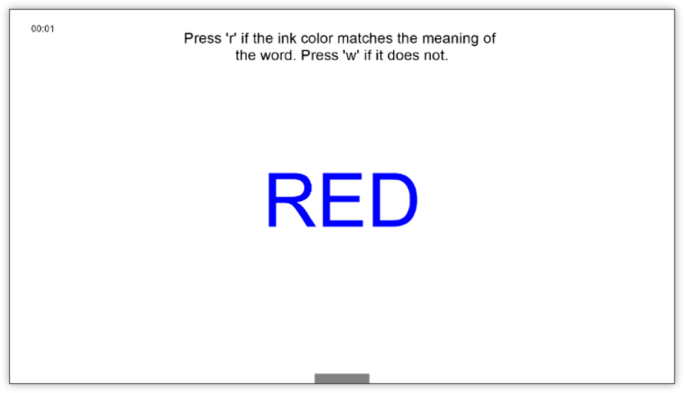
Example, where the ink color does not match the word meaning.

#### Questionnaire data

Within the study, we will ask the participants to answer four different questionnaires *Q0*, *Q1B/Q1I*, *Q2*, and *BF-10*. *Q0* will be administered at the beginning of the study to characterize the study participants by collecting demographic variables such as gender, age, language level, and music preferences. Music preferences will be self-assessed through a multiple-choice option. If the favorite music genre, or the music genre actually used for work, is not present, participants can specify their preferences in a free-text field. In addition, the standardized *BF-10* will be used to determine the personality based on the five-factor model [35], [36]. *Q1B* will be asked after every phase to get information about the self-assessed stress and concentration level measured through a visual slider. If the phase was an intervention, the questionnaire *Q1B* would ask additionally if the participant knew the song before (yes/no/unsure) and if they liked the song (visual slider). At the end of the study, participants are asked to complete *Q2* to receive information about their personal beliefs, whether the intervention affected their concentration or not. A complete list of the questionnaires and their questions can be found in Supplementary Text 1.

#### Sensor data

In addition to questionnaire data, we will collect biomedical sensor data. We will collect continuous Electroencephalography (EEG) measurements with the *Emotiv Insight 2* sensor to assess the electrical activity of the brain and estimate, through that, the cognitive load of the individual during the phases [37]. An eye tracking sensor collects information about the movement of the eyes and the pupil’s diameter to evaluate mental stress within the study [38]. In this study, the *Tobii Pro Nano* sensor is used. Electrodermal Activity (EDA) data will be used to investigate the skin conductance, which is associated with cognitive load and stress level [5]. We collect Heart Rate Variability (HRV) and Electrodermal Activity (EDA) data through the *Shimmer3 GSR+* sensor, which can complement the analytics of the EDA data for physiological stress response [39]. The collection of diverse biomedical sensor data will enable the creation of a rich dataset to assess stress via physiological signals. The multimodal approach of collecting measurements from various sensors can help analyze cognitive load and stress. The downsides of one modality, such as the sensitivity of the pupil size to lighting conditions, can be compensated by other modalities.

The collection of sensor data is entirely voluntary, and participants may choose to opt out without affecting their participation in other parts of the study. The data will be collected through the Lab Streaming Layer (LSL) as an open-source network middleware ecosystem [40]. It will collect and synchronize the streamed sensor data. Further, a *python* script will be used to collect questionnaire data and track responses of the Stroop task. Data collected before or after the intervention and control phase, e.g., during questionnaires, will be deleted right after the recording and will not be used in any analysis.

### 2.4 Participant timeline

The study will be conducted in certain steps explained in the following. An overview of the schedule of enrollment, allocation, study periods, and close-out can be found in Supplementary Table 2.

#### Study registration

Interested participants schedule an initial meeting through direct contact via email. Available time slots for onsite participation are shared, and an appointment will be scheduled.

#### Consent to study

At the beginning of the scheduled session, participants will receive a concise explanation of the study’s purpose, procedures, potential risks and benefits, data handling policies, and their right to withdraw at any time without penalty. Any questions will be addressed before participants sign the informed consent form, confirming their voluntary participation and understanding of the study. A copy of the signed consent form will be provided to each participant, and the original will be securely stored.

#### Conducting the trial

Participants will proceed with the trial, following the randomized study design explained in Section 2.2. The use of sensors is entirely voluntary, and participants may choose to opt out without affecting their participation in other parts of the study. Each trial session lasts approximately 20 min, including breaks between testing phases.

#### Discussing results and feedback session

Upon completing the trial, participants receive an overview of their preliminary results, including the preliminary results of the individual Bayesian analysis described in detail in Section 2.5. A brief feedback session allows participants to share their experiences, preferences, and impressions of the study.

### 2.5 Statistical methods

#### Estimand

In order to define the estimand of interest, we specify the population, intervention, endpoint, summary, and intercurrent events following the ICH-E9 (R1) statement for estimands. The population includes all enrolled participants who start the trial. The participants are exposed in the intervention condition to music from their self-selected music genre. In the control condition, no music will be played. The endpoint is the binary outcome of each Stroop trial (correct vs. incorrect), measured repeatedly within the periods. For each participant and condition, we will summarize performance as the proportion of correct responses. The estimand of interest is the population-level average effect of music on concentration, estimated as the average across participants of the within-participant differences in the Stroop proportions between music and no-music periods. Additionally, we are interested in individual-level intervention effects, estimated as within-participant differences in the primary outcome between music and no music conditions. Missed responses are counted as incorrect; besides that, no intercurrent events are expected.

#### Primary analysis

To estimate the population-level estimand of interest, we will use a Bayesian generalized linear mixed model (GLMM). The model will include a fixed effect for condition (music vs. no music) and a participant-specific random intercept to account for baseline differences in performance. Hence, it will account for the repeated binary outcomes within participants and individual differences in baseline performance. We will use weakly informative priors to regularize estimates and make prior assumptions explicit. The posterior distribution of intervention effect will be summarized by posterior means/medians, 95% credible intervals, and posterior probabilities (e.g., $\mathrm{Pr}\left(\beta_{1}>0|\text{data}\right)$). Marginal risk differences and odds ratios will be derived from posterior predictive simulations to facilitate interpretation on both the probability and odds scales.

More formally, let $y_{ij}=1$ denote the correct answer in the Stroop task j for individual i and $y_{ij}=0$ denote a wrong answer, respectively. This will be drawn from a Bernoulli distribution with the corresponding probability $p_{ij}$:

(3) $$y_{ij}∼\text{Bernoulli}\left(p_{ij}\right)$$

We will use a logistic link, and a fixed effect for the condition $A_{ij}$, where $A_{ij}=1$ denotes listening to music and $A_{ij}=0$ denotes not listening to music, respectively. Further, we will include a random intercept $b_{i}∼N\left(0,\sigma_{b}^{2}\right)$ for each participant i drawn from a normal distribution.

(4) $$\text{logit}\left(p_{ij}\right)=\beta_{0}+\beta_{1}\cdot {\text{A}}_{ij}+b_{i}$$

We will use non-informative Priors for the β coefficients following a normal distribution and for $\sigma_{b}^{2}$ a half-student-t distribution with a degree of freedom of 3 and a standard deviation of 1. For the intercept $\beta_{0}$, we choose a large standard deviation to allow a high flexibility:

(5) $$\beta_{0}∼N\left(0,5^{2}\right)$$

$$\beta_{1}∼N\left(0,1\right)$$

$$\sigma_{b}^{2}∼\text{Half-student-t}\left(v=3,\sigma^{2}=1\right)$$

To assess the robustness of the findings, sensitivity analyses are planned. First, as a non-parametric robustness check, we will perform Fisher’s exact test on aggregated counts of correct versus incorrect responses under music and no-music conditions, which provides a simple benchmark for differences between music and no-music conditions, assuming independence of observations. Next, we will include chronological time in the Bayesian generalized linear mixed model (GLMM) with uninformative priors ($\beta_{i}∼N\left(0,1\right)$), as a proxy for time-varying effects of factors such as fatigue or learning. From the fitted models, we will report odds ratios with 95% credible intervals and compute average marginal risk differences for interoperability.

If a value of $y_{ij}$ is missing (e.g., the participant did not respond within the given time frame), it will count as incorrect and be coded as 0. In the case that multiple consecutive values are missing, the length of the missing data will be reported.

In addition to the population-level estimates, we will estimate individual-level intervention effects by contrasting within-person differences in accuracy between music and no-music conditions for each participant across their trial periods. To illustrate this, we will follow the Independent Bayes Approach (IBA) described in Hoffmann et al. [41], presenting participant-specific estimates, visualizations, and posterior probabilities of benefit where appropriate. These analyses will help to characterize heterogeneity in individual responses to the intervention. For the analysis, we will use aggregated counts of the correct answers of the Stroop task.

#### Secondary analysis

To address the first secondary question, to what extent physiological sensor data can serve as proxies for concentration changes, as measured by the cognitive task, we will use descriptive and visual analytics. Time-series plots, condition-wise summaries, and participant-level comparisons will be generated to explore signal patterns across the intervention and control phases. If sensor data is missing, we will not impute the data and report only cases where we observe the sensor data for at least 60 consecutive seconds in every period. Summary statistics such as means, standard deviations, medians, and interquartile ranges will be computed for each physiological signal, stratified by condition (music vs. no music) and participant. Visualizations may include line plots, box plots, and heat maps to explore temporal dynamics and individual differences. Furthermore, methods from time series analysis will be applied to detect changes before and after incorrect answers, thereby elaborating on the stress and concentration levels in relation to the given answer within this time frame.

To explore the second secondary research question, the extent to which individual differences such as music preference or playing an instrument moderate the effect of music on cognitive task performance, we will first stratify the descriptive statistics described in Section 2.5 for different subgroups. Subgroup analyses will be conducted only for groups that include at least five participants meeting the specific criteria. First, we will investigate listening behavior and music preferences. For this analysis, we will distinguish between participants who often or always listen to music at work and those who listen only occasionally, rarely, or never. Next, we will investigate the subgroup of participants who have ever played an instrument (Answers *Yes* and *Not anymore*). Finally, we will include these variables of interest with uninformative priors (β∼N(0,1)) as interaction effects in the Bayesian generalized linear mixed model (GLMM) used for the primary research question. We will adjust the model for the individual characteristics and experience and report the estimated effect and its estimated 95% credible interval for each model.

#### Interim analysis

Immediately after the end of each trial, individual descriptive statistics will be calculated and reported to the participant, who can discuss them with the study conductor. This report presents the absolute and relative numbers of correctly classified answers, represented in a contingency table, as well as the average time spent answering the task in each period. Neither the sensor data nor the questionnaire data will be analyzed in the interim analysis. For the individual-level analyses, credible intervals will be computed. No p-values nor confidence intervals will be calculated on an individual level at this stage. The study participants will be informed that they should not communicate the results to others until the study is completed, to avoid interference, i.e., influencing trials of other participants.

### 2.6 Data handling

#### Collection and management

Before participating in the study, each participant will receive detailed information about data collection, storage, and deletion protocols. Participants will also be informed that study data will be published anonymously, linked only to a randomly generated pseudonym.

Study data will be collected on-site using Python and stored anonymously on local devices at the institution, with no retention of names, email addresses, or other personal data. A random pseudonym will be generated and provided to each participant, allowing only the participant to re-identify their own data. This is part of an irreversible anonymization process to ensure data security and privacy.

The data can only be deleted at the in-person meeting, as after the in-person meeting, the data cannot be retracted.

The data will be analyzed by trained investigators from the institute and the university. After the study is finished, anonymized data as well as the code base for conducting the experiment will be published online under the CC BY-NC license. The code will be published through GitHub, and the data through a suitable data sharing platform, along with the data description. The data sharing statement can be found in Table 3.

**Table 3:** Data sharing statement.

Element	Attribute
Will individual participant data be available?	Yes

What data in particular will be shared?	All of the anonymized individual participant data, including questionnaires and sensor data if possible, will be shared

What other documents will be available?	Study Protocol, Analytical Code

When will the data be available?	Immediately after the publication, without an end date.

With whom?	Anyone who wished to access the data

What type of analysis?	Any non-commercial purpose (CC BY-NC license)

By what mechanism will data be made available?	An online repository will be set up

#### Monitoring

In this study, the data will be collected automatically through the Python code base and the Lab Streaming Layer. After each phase, the sensors will be checked for failures, and at the end of each individual trial, the data will be summarized automatically to double-check the start and end of measurements, the number of observations for each sensor, and the number of tracked responses of the Stroop task. These reports will be stored and used for data documentation. Further monitoring and audit are not planned.

#### Adverse event reporting and harms

The safety and well-being of the study participants are a priority throughout the study. Participants will be monitored in person during on-site sessions to ensure their comfort and to allow for immediate communication of any concerns or adverse events, although no adverse events are expected given the nature of the intervention. Listening to music is considered a low-risk activity and is not associated with known physical or psychological hazards. In the unlikely case of an adverse event, the investigator, who is trained in first aid, will provide immediate assistance. Additionally, the study will adhere to the safety protocols and emergency procedures established by the campus, ensuring prompt and appropriate action in case of any unforeseen circumstances. Participants will be informed about the minimal risks associated with the study and will have the opportunity to withdraw at any time. The study does not involve interventions that are invasive or potentially harmful, and no long-term follow-up for safety is deemed necessary. During the trial, we expect the participant to focus on a concentration and attention task, and look at a screen. That can lead to a feeling of tiredness, exhaustion, and might lead to a slight headache. The tasks will be performed under time pressure, so the study participant might feel stressed.

#### Auditing

No formal external auditing is planned for this aggregated N-of-1 trial. Internal monitoring will be conducted by the principal investigator, who is not involved in daily data collection. This includes verifying that all sessions are conducted according to the protocol, data files are complete and correctly labeled, and any deviations are documented. Automated scripts will perform data integrity checks (e.g., sampling rates, missing data) after each session and report the results of those checks.

### 2.7 Dissemination

#### Protocol amendments

Any modifications to the protocol that may impact the conduct of the study, the potential benefit or risk to participants, or the scientific validity of the trial (including changes to eligibility criteria, study design, outcomes, analyses, or procedures) will require a formal amendment. Amendments will be documented with a new protocol version number and date, and will be submitted to the relevant ethics committee for approval before implementation. All amendments will also be updated in the trial registry record and in any open repositories where the protocol is hosted. Minor administrative changes (e.g., correction of typographical errors) will be documented in the protocol history but will not require ethics notification.

#### Confidentiality

All participant information will be handled in accordance with applicable data protection regulations. Personal identifiers on the study consent will be stored separately from research data and replaced with a unique study ID. There will be no linking of the participant identities to study IDs. Raw data from the Stroop test and physiological sensors will be stored on encrypted devices and transferred to secure institutional servers. Access to the data will be restricted to authorized study personnel. Anonymized datasets will be used for all analyses and will be made publicly available only in non-identifiable form. All physical documents (e.g., consent forms) will be stored in locked cabinets in restricted-access offices. Data will be retained for a maximum of 3 years after study completion, after which it will be securely deleted or destroyed.

#### Access to data

Only the principal investigator, designated co-investigators, and trained scientific staff will have access to identifiable participant data during the study. All such individuals will receive appropriate training in data protection and confidentiality before handling any data. An anonymized version of the final dataset, along with the analysis code and study materials, will be made publicly available in an open-access repository upon publication of the primary results. Access to identifiable data will not be granted to any third party without explicit consent from the participant and approval from the relevant ethics committee.

#### Ancillary and post-trial care

No ancillary or post-trial care will be provided to participants upon completion of the study. Participants will be informed of this policy before enrolling in the trial. The decision not to offer post-trial care is based on the nature and scope of the study.

#### Dissemination policy

The findings of this study will be submitted for publication in a peer-reviewed open-access journal and presented at relevant scientific conferences. The full protocol, anonymized dataset, and analysis code will be made publicly available via an open repository within 36 months after study completion, under a Creative Commons Attribution (CC BY-NC 4.0) license.

## 3 Discussion

This study leverages the N-of-1 trial design to provide a personalized approach to understanding the effects of self-selected music genre on concentration. Unlike traditional group-based studies that average effects across participants, the N-of-1 trial design allows to include a personalized protocol for each participant, the efficient estimation of population-level treatment effects as well as of individual-level treatment effects, thereby addressing the substantial variability in how music influences cognitive performance. Although the effect of music on concentration has been explored in numerous studies, results remain inconsistent and inconclusive, likely due to the high degree of individual variability and differences in study designs. By combining a rigorous N-of-1 trial design with repeated measures, this study contributes to the understanding of personalized cognitive responses.

In this study, the participant can select a music genre of their choice. With that, the study allows adjustment for personal preferences. However, the selection of music genres is broader compared to artist or track choice. While other studies have used white noise, office noise, or speech content as the baseline condition, this study aims to mimic a silent work environment without music or additional noise as a comparator. While the study will be conducted in a separate room on the university campus, the study setup does not guarantee complete silence.

The digital adaptation of the Stroop test is employed as an outcome assessment tool to evaluate concentration. While this test provides a practical and flexible measure, it has limitations, particularly in its lack of heavy standardization within the framework of music and cognitive studies. These limitations should be acknowledged when interpreting results, as they may affect the comparability of findings across similar studies.

The sample size calculation for this study is based on generalized linear mixed model (GLMM). The assumed values are based on pilot testing with limited prior evidence and may not fully reflect the true effect size observed during the study. Further, we powered for a small aggregated treatment effect difference of 95% for the no-music condition and 96% for the music condition, which may not be clinically relevant for all participants. Despite this, the individualized nature of the study allows for meaningful intra-participant comparisons, even in the context of such uncertainties.

In this study, the treatment allocation sequence is randomized separately for each participant. Prior to the experimental period, participants will complete seven practice Stroop tasks: two without a time limit and five with a time limit, to familiarize themselves with the task and reduce learning effects during the intervention periods. Washout phases are included between treatment periods to minimize potential carryover effects.

Based on this design, major sequence effects or carryover are not expected. Nevertheless, residual time-related effects such as learning or fatigue cannot be fully excluded. Therefore, although the primary analysis does not explicitly adjust for treatment sequence, sensitivity analyses will explore the robustness of the results with respect to possible temporal trends.

In summary, this study not only explores a novel personalized approach to understanding the effects of music on concentration but also highlights the importance of individualized methodologies in cognitive research. While acknowledging the limitations of the outcome assessment tool, the study provides a valuable framework for future investigations into personalized cognitive enhancement strategies. In addition to advancing our understanding of how music impacts concentration, the study will contribute to the research community by publishing the collected data online. This open-access dataset could serve as a benchmark for developing and testing statistical methodologies for N-of-1 trials, fostering further advancements in personalized cognitive research. Furthermore, the integration of sensor data in secondary analyses will offer unique insights into the physiological correlates of concentration and the potential influence of music on these multimodal measures. Together, these contributions underscore the study’s potential to inform both theoretical understanding and practical applications in personalized cognitive enhancement.

## CRediT authorship contribution statement

**Thomas Gärtner:** Writing – original draft, Visualization, Validation, Software, Resources, Methodology, Investigation, Formal analysis, Conceptualization. **Fabian Stolp:** Writing – review & editing, Software, Conceptualization. **Bert Arnrich:** Writing – review & editing, Resources . **Stefan Konigorski:** Writing – review & editing, Supervision, Resources, Project administration.

## Trial registration

This trial is registered at German Clinical Trial Register, identifier DRKS00038295.

## Ethical approval

All participants will provide informed consent, and the study will be conducted in accordance with the Declaration of Helsinki. It was approved by the Ethics Committee of the University of Potsdam, approval number 71/2025.

### Study status

Study recruitment is planned to start in December 2025, with an expected end in March 2026; study completion is expected in July 2026.

## Declaration of Generative AI and AI-assisted technologies in the writing process

During the preparation of this work, the authors used GPT.UP and Grammarly to improve the language and readability. After using these tools, the authors reviewed and edited the content as needed and take full responsibility for the content of the publication.

## Declaration of competing interest

The authors declare that they have no known competing financial interests or personal relationships that could have appeared to influence the work reported in this paper.

**Funding** Open Access funding enabled and organized by Projekt DEAL. Funded by the Deutsche Forschungsgemeinschaft (DFG, German Research Foundation) – project number 491466077.

## Data Availability

No data was used for the research described in the article.

## Appendix A Supplementary data

MMC S1 Supplementary material contains the participant information materials used in the study. MMC S1
