# Supplementary material for: Study protocol for PIANo-1: Personalized Investigation of music’s effect on Attention in a series of N-of-1 trials
Source: Contemp Clin Trials Commun. 2026 Jan 22;49:101606. doi: 10.1016/j.conctc.2026.101606 (PMC12873732; doi:10.1016/j.conctc.2026.101606)
Supplement: MMC S1 — Supplementary material contains the participant information materials used in the study. [file mmc1.pdf]

Supplemental Material
Study Protocol for PIANo-1:
Personalized **I**nvestigation of Music's Effect on **A**ttention in a Series of **N**-**o**f-1 trials

Anonymized

December 8, 2025

Supplementary Tables 1 Table of Songs

Genre	Title	Artist	Lyrics	Length	Tempo (bpm)	Note	Key
Alternative-Indie	Photosynthese	Dilla	German	3:20	168	G	Major
Alternative-Indie	Pocahontas	Annenmaykantereit	German	3:05	119	D	Minor
Alternative-Indie	Dog Days Are Over	Florence+the machine	English	4:11	150	G	Major
Alternative-Indie	L.O.V.E.	Leoniden	English	3:14	150	F♯/G♭	Minor
Ambient	Echoes	Leya Watson	No Lyrics	3:29	138	G	Major
Ambient	Weightless	Marconi Union	No Lyrics	8:00	71	D	Minor
Cinematic	Very Old Friend (Lord of the Rings)	Howard Shore	No Lyrics	3:12	76	G	Major
Cinematic	Main Theme (from Jurassic Park)	John Williams	No Lyrics	5:29	133	A♯/B♭	Major
Classic	Prelude and Fugue No- 1 in C Major	Johann Sebastian Bach	No Lyrics	4:27	112	C	Major
Classic	Nocturne Op.9 No.2	Frédéric Chopin	No Lyrics	5:02	138	A♯/B♭	Major
Classic-Rock	Sweet Child O' Mine	Guns N' Roses	English	5:56	125	F♯/G♭	Major
Classic-Rock	Dancing in the dark	Bruce Springsteen	English	4:01	149	C♯/D♭	Minor
EDM	Animals	Martin Garrix	No Lyrics	5:04	128	C♯/D♭	Major
EDM	More Intensity	Daniel Portman	No Lyrics	3:39	127	C♯/D♭	Major
Folk	Stubborn Love	The Lumineers	English	4:39	115	C	Major
Folk	Home	Edward Sharpe & The Magnetic Zeros	English	5:03	112	D	Major
Hip-Hop	Rapper's Delight	The Sugarhill Gang	English	3:55	112	D	Major
Hip-Hop	Planet Rock	Afrika Bambaataa & the Soul Sonic Force	English	5:19	127	F♯/G♭	Minor
K-Pop	FAKE LOVE	BTS	English	4:02	78	D	Minor
K-Pop	Gee	Girls Generation	English	3:21	100	C♯/D♭	Minor
LoFi-Jazz	Seedless Grape	Kredylen	No Lyrics	3:20	79	G♯/A♭	Major
LoFi-Jazz	Shabba Doo	Chakra Blue	No Lyrics	3:19	81	D♯/E♭	Major
Metal	Chop Suey!	System of a down	English	3:30	127	G	Minor
Metal	Enter Sandman	Metallica	English	5:31	123	F♯/G♭	Minor
Pop	Solange du dich bewegst	Wilhelmine	German	3:31	120	A♯/B♭	Major
Pop	Chöre	Mark Forster	German	3:28	130	F♯/G♭	Minor
Pop	Shake It Off (Taylor's Version)	Taylor Swift	English	3:39	160	G	Major
Pop	Golden	Harry Styles	English	3:28	140	E	Minor
Rock	Alles auf Rausch	Feine Sahne Fischfilet	German	3:16	181	C	Major
Rock	Schrei nach Liebe	Die Ärzte	German	4:12	161	D	Minor
Rock	Can't Stop	Red Hot Chili Peppers	English	4:29	91	A	Major
Rock	Learn to Fly	Foo Fighters	English	3:55	136	E	Major
Techno	A New Error	Moderat	No Lyrics	6:07	111	B	Minor
Techno	Around the World	Daft Punk	No Lyrics	7:09	121	G	Major

Supplementary Tables 2 SPIRIT Figure

**Timepoint** *	Enrollment	Allocation	**Study Period**				Close-out
	t_{-1}	t_0	P_1	P_2	P_3	P_4	$\mathbf{t_x}$
**Enrollment:**							
Eligibility screen	×						
Informed consent	×						
Allocation		×					
**Interventions (illustrating ABAB):**							
Listening to music			×		×		
[Baseline: Do not listen to music]				×		×	
**Assessments:**							
Assess Baseline Questionnaire Q0		×					
Assess Concentration with Stroop Test			×	×	×	×	
Assess Questionnaire Q1I			×		×		
Assess Questionnaire Q1B				×		×	
Collect Sensor Data			×	×	×	×	
Assess Questionnaire Q2							×

* Time points are referring to the start of the trial t_0, t_{-1} to a time point directly before the study, P indicates the study period and t_x the end of the study.

Table 2: Schedule of enrollment, interventions, and assessments visualizing the study for *ABAB* treatment allocation.

Supplementary Tables 3 SPIRIT Checklist with SPENT extension

**Title**	**Item**	**SPENT extension**	**Page**
**Section 1: Administrative Data**			
Title	1a	Descriptive title, including "N-of-1 trial" and “protocol”. For series: Descriptive title, including "a series of N-of-1 trials" and “protocol”.	Title
	1b	For specific guidance on abstracts, see SPENT Guidance for Abstracts. (Appendix table 2).	Abstract
Trial Registration	2a	Trial identifier and registry name. If not yet registered, name of intended registry.	Section 3
	2b	All items from the World Health Organization Trial Registration Data Set. (WHOTRDS).	See GCTR, Section 3
Protocol version	3	Date and version identifier	
Funding	4	Sources and types of financial, material, and other support.	Section 3
Roles and responsibilities	5a	Names, affiliations, and roles of protocol contributors.	Section 3
	5b	Name and contact information for the trial sponsor.	Section 3
	5c	Role of study sponsor and funders, if any, in study design; collection, management, analysis, and interpretation of data; writing of the report; and the decision to submit the report for publication, including whether they will have ultimate authority over any of these activities.	Section 3
	5d	Composition, roles, and responsibilities of the coordinating center, steering committee, end point adjudication committee, data management team, and other individuals or groups overseeing the trial, if applicable. (see Item 21a for DMC)	*Not applicable.*
**Section 2: Introduction**			
Background and rationale	6a	Description of research question and justification for undertaking the trial, including summary of relevant studies (published and unpublished) examining benefits and harms for each intervention, and rationale for using N-of-1	Section 1
	6b	Explanation for choice of comparators.	Section 1
Objectives	7	Specific objectives or hypotheses.	Section 1.1
Trial design	8	Description of the trial design, including N-of-1 trial or series of trials, and framework (eg, superiority, equivalence, non-inferiority, exploratory). In addition for series: Explanation of the series design including whether the design will be tailored to each participant.	Section 1.2
**Section 3: Methods**			
Participants, interventions, and outcomes			
Study Setting	9	Description of study settings (eg, community clinic, academic hospital) and list of countries where data will be collected. Reference to where list of study sites can be obtained.	Section 2.1

Continued on next page

Title	Item	SPENT extension	Page
Eligibility criteria	10	Inclusion and exclusion criteria for participants. If applicable, eligibility criteria for study centers and individuals who will perform the interventions (eg, surgeons, psychotherapists). Diagnosis/disorder, diagnostic criteria, co-morbid conditions and concurrent therapies. For series: Same as SPIRIT item 10	Section 2.1
Interventions	11a	Intervention(s) for each period with sufficient detail to allow replication, including how and when they will be administered, planned number of periods, and duration of each period (including run-in and washout, if applicable). In addition for series: How the design will be tailored to each participant, if applicable	Section 2.2
	11b	Criteria for discontinuing or modifying allocated interventions for a given trial participant (eg, drug dose change in response to harms, participant request, or improving/ worsening disease).	Section 2.2
	11c	Strategies to improve adherence to intervention protocols, and any procedures for monitoring adherence (eg, drug tablet return, laboratory tests).	Section 2.2
	11d	Relevant concomitant care and interventions that are permitted or prohibited during the trial.	Section 2.2
Outcomes	12	Primary, secondary, and other outcomes, including the specific measurement variable (eg, systolic blood pressure), analysis metric (eg, change from baseline, final value, time to event), method of aggregation (eg, median, proportion), and time point for each outcome. Explanation of the clinical relevance of chosen efficacy and harm outcomes is strongly recommended	Section 2.3
Participant timeline	13	Time schedule of enrollment, interventions (including any run-ins and washouts), assessments, and visits for participants. A schematic diagram is highly recommended (Figure).	Section 2.4, Supplementary Table 2
Sample size	14	Estimated number of intervention periods and measurements/observations needed to achieve study objectives within an individual N-of-1 trial. In addition for series: Estimated number of participants needed to achieve study objectives. How these numbers were determined, including clinical and statistical assumptions supporting any sample size calculations.	Section 2.1
Recruitment	15	Strategies for achieving adequate participant enrollment to reach target sample size. For series: strategies for achieving adequate participant enrollment to reach target sample size	Section 2.1
Assignment of interventions (for controlled trials)			
Allocation - sequence generation	16a	Method of generating the allocation sequence (eg, computer-generated random numbers), and list of any factors for stratification. To reduce predictability, details of any restrictions (eg, pairs, blocking) should be provided in a separate document that is unavailable to those who enrol participants or assign interventions. In addition for series: List of any factors for stratification.	Section 2.2

Continued on next page

Title	Item	SPENT extension	Page
- concealment mechanism	16b	Mechanism of implementing the allocation sequence (eg, central telephone; sequentially numbered, opaque, sealed envelopes), describing any steps to conceal the sequence until interventions are assigned.	Section 2.2
- implementation	16c	Who will generate the allocation sequence, who will enroll participants, and who will assign participants to interventions.	Section 2.2
Blinding (masking)	17a	Who will be blinded after assignment to interventions (eg, trial participants, care providers, outcome assessors, data analysts), and how.	Section 2.2
	17b	If blinded, circumstances under which unblinding is permissible, and procedure for revealing a participant's allocated intervention during the trial.	Section 2.2
Data collection, management, and analysis			
Data collection methods	18a	Plans for assessment and collection of outcome, baseline, and other trial data, including any related processes to promote data quality (eg, duplicate measurements, training of assessors) and a description of study instruments (eg, questionnaires, laboratory tests) along with their reliability and validity, if known. Reference to where data collection forms can be found, if not in the protocol.	Section 2.6
	18b	Plans to promote participant retention and complete follow-up, including list of any outcome data to be collected for participants who discontinue or deviate from intervention protocols	Section 2.6
Data management	19	Plans for data entry, coding, security, and storage, including any related processes to promote data quality (eg, double data entry; range checks for data values). Reference to where details of data management procedures can be found, if not in the protocol.	Section 2.6
Statistical methods	20a1	Statistical methods for analyzing primary and secondary outcomes for each individual. Reference to where other details of the statistical analysis plan can be found, if not in the protocol. In addition for series: if planned, proposed methods of quantitative synthesis of individual trial data, and how heterogeneity between participants will be assessed	Section 2.5
	20a2	Statistical methods to account for correlation introduced by the repeated measures and crossover design of N-of-1 studies.	Section 2.5
	20b	Methods for any additional analyses (eg, subgroup and adjusted analyses).	Section 2.5
	20c	Statistical methods to handle missing data (eg, multiple imputation, modelling). In addition for series: Definition of analysis population relating to protocol non-adherence (eg, as-randomized analysis).	Section 2.5
Monitoring			
Data monitoring	21a	Composition of Data Monitoring Committee (DMC); summary of its role and reporting structure; statement of whether it is independent from the sponsor and competing interests; and reference to where further details about its charter can be found, if not in the protocol. Alternatively, an explanation of why a DMC is not needed	Section 2.6

Continued on next page

Title	Item	SPENT extension	Page
	21b	Description of any interim analyses and stopping guidelines, including who will have access to these interim results and make the final decision to terminate the trial.	Section 2.6
Harms	22	Plans for collecting, assessing, reporting, and managing solicited and spontaneously reported adverse events and other unintended effects of trial interventions or trial conduct	Section 2.6
Auditing	23	Frequency and procedures for auditing trial conduct, if any, and whether the process will be independent from investigators and the sponsor	Section 2.6
**Section 4: Ethics & Dissemination**			
Research ethics approval	24	Plans for seeking REC/IRB approval.	Section 3
Protocol amendments	25	Plans for communicating important protocol modifications (eg, changes to eligibility criteria, outcomes, analyses) to relevant parties (eg, investigators, RECs/IRBs, trial participants, trial registries, journals, regulators).	Section 2.7
Consent or assent	26a	Who will obtain informed consent or assent from potential trial participants or authorized surrogates, and how (see item 32).	Section 2.4
	26b	Additional consent provisions for collection and use of participant data and biological specimens in ancillary studies, if applicable.	Section 2.4
Confidentiality	27	How personal information about potential and enrolled participants will be collected, shared, and maintained in order to protect confidentiality before, during, and after the trial.	Section 2.7
Declaration of interests	28	Financial and other competing interests for principal investigators for the overall trial and each study site	Section 3
Access to data	29	Statement of who will have access to the final trial data set, and disclosure of contractual agreements that limit such access for investigators	Section 2.7
Ancillary and post-trial care	30	Provision, if any, for ancillary and post-trial care, and for compensation to those who suffer harm from trial participation.	Section 2.7
Dissemination policy	31a	Plans for investigators to communicate each individual's results to the participant. Plans for investigators and sponsor to communicate trial results to participants, health care professionals, the public, and other relevant groups (eg, via publication, reporting in results databases, or other data-sharing arrangements), including any publication restrictions	Section 2.7
	31b	Authorship eligibility guidelines and any intended use of professional writers.	Section 2.7
	31c	Plans, if any, for granting public access to the full protocol, participant-level data set, and statistical code.	Section 2.7
**Section 5: Appendices**			
Informed consent materials	32	Model consent form, and other related documentation were given to participants and authorized surrogates.	Supplementary Text 2
Biological specimens	33	Plans for collection, laboratory evaluation, and storage of biological specimens for genetic or molecular analysis in the current trial and for future use in ancillary studies, if applicable.	*Not Applicable.*

Supplementary Tables 4 Checklist for Reporting Guidelines for Music-based Interventions

Item	Title	Description	Section
1	Brief Name	Provide the name or phrase that describes the intervention	Section 1.1
2	Intervention Theory and/or Scientific Rationale	Provide a rationale for the music and/or music experience(s). Specify how essential features of the music and music experience(s) are expected to influence targeted outcomes	Section 1
3a	Music Selection	Describe the process for how music was selected, including who was involved in music selection.	Section 2.2
3b	Music	Specify key details about the music that may be relevant to specified outcomes of interest. Characteristics may include compositional features of the music (such as tempo, harmony, rhythm, pitch, tonality, form, instrumentation), sound intensity or volume, lyrics, and/or how the music relates to the participants' cultural identity and heritage. When using published music, provide a reference for a sound recording or sheet music.	Section 2.2, Supplementary Table 1
3c	Music delivery method	Provide details about how music was provided to or created with participants (such as live, recorded, computer generated). Include any details necessary for replication. This might include size of performing group, use of playback equipment, or person controlling volume.	Section 2.2
3d	Materials	List all materials necessary for the music experience. Include music and non-music equipment and materials.	Section 2.1
3e	Intervention Strategies	Describe the music intervention strategy or strategies being studied (such as music listening, improvisation, song writing, rhythmic auditory stimulation).	Section 2.2
4	Interventionist	Specify interventionist qualification, credentials, training, and/or experience. Indicate how many interventionists deliver the music experience.	Section 2.2
5	Individual or Group Intervention	Specify whether interventions were delivered to individuals or groups of individuals. For group interventions, specify the size of the group.	Section 2.2
6	Setting	Describe where the intervention was delivered. Include location, privacy level, ambient sound, and/or any other factors that may have affected participants' experiences.	Section 2.1
7	Intervention Delivery Schedule	Report number of sessions, session length (for example, 60 min), frequency (for example, $3\times$/week)	Section 1.2
8	Treatment Fidelity	Describe strategies and/or measures used to ensure that the music intervention was delivered and received as intended.	Section 2.6

Supplementary Text 1 Questionnaires

Supplementary Text 1.1 Questionnaire BF10

- 1 - I see myself as someone who is reserved. (choice)
  - Disagree strongly
  - Disagree a little
  - Neither agree nor disagree
  - Agree a little
  - Agree strongly
- 2 - I see myself as someone who is generally trusting. (choice)
  - Disagree strongly
  - Disagree a little
  - Neither agree nor disagree
  - Agree a little
  - Agree strongly
- 3 - I see myself as someone who tends to be lazy. (choice)
  - Disagree strongly
  - Disagree a little
  - Neither agree nor disagree
  - Agree a little
  - Agree strongly
- 4 - I see myself as someone who is relaxed, handles stress well. (choice)
  - Disagree strongly
  - Disagree a little
  - Neither agree nor disagree
  - Agree a little
  - Agree strongly
- 5 - I see myself as someone who has few artistic interests. (choice)
  - Disagree strongly
  - Disagree a little
  - Neither agree nor disagree
  - Agree a little
  - Agree strongly
- 6 - I see myself as someone who is outgoing, sociable. (choice)
  - Disagree strongly
  - Disagree a little
  - Neither agree nor disagree
  - Agree a little
  - Agree strongly

- 7 - I see myself as someone who tends to find fault with others. (choice)
  - Disagree strongly
  - Disagree a little
  - Neither agree nor disagree
  - Agree a little
  - Agree strongly
- 8 - I see myself as someone who does a thorough job. (choice)
  - Disagree strongly
  - Disagree a little
  - Neither agree nor disagree
  - Agree a little
  - Agree strongly
- 9 - I see myself as someone who gets nervous easily. (choice)
  - Disagree strongly
  - Disagree a little
  - Neither agree nor disagree
  - Agree a little
  - Agree strongly
- 10 - I see myself as someone who has an active imagination. (choice)
  - Disagree strongly
  - Disagree a little
  - Neither agree nor disagree
  - Agree a little
  - Agree strongly

Supplementary Text 1.2 Questionnaire Q0

- 1 - General Questions (heading)
- 2 - What is your gender identity? (choice)
  - Female
  - Male
  - Non-Binary
  - Prefer not to say
- 3 - What is your age? (choice)
  - 18-25
  - 26-30
  - 31-40
  - 41-50
  - 51-60
  - >60

  - Prefer not to say

- 4 - What is your proficiency with English? (choice)
  - Native
  - Fluent
  - Advanced
  - Beginner
  - No
- 5 - What is your proficiency with German? (choice)
  - Native
  - Fluent
  - Advanced
  - Beginner
  - No
- 6 - What is your physical workload in your daily work? (slider)
- 7 - What is your mental workload in your daily work? (slider)
- 8 - Music (heading)
- 9 - Do you prefer listening to music with lyrics or instrumental music? (choice)
  - Only with lyrics
  - Mostly with lyrics
  - Mix of both
  - Mostly instrumental
  - Only instrumental
  - No preference
- 10a - How often do you listen to music? (choice)
  - Always
  - Often
  - Occasionally
  - Rarely
  - Never
- 10b - Which of the following music genres do you like most? (choice)
  - Afrobeats
  - Alternative/Indie
  - Ambient
  - Blues
  - Classic Rock
  - Classical
  - Country
  - EDM

  - Film Score
  - Folk
  - Hip-Hop/Rap
  - House
  - Jazz
  - K-Pop
  - Latin/Reggaeton
  - LoFi
  - Metal
  - Pop
  - R&B/Soul
  - Reggae
  - Rock
  - Swing
  - Techno
  - Other

- 10c - Which of the following music genres do you like most - Could you specify 'Other' please: (free text - only if 10b include Other)

- 11a - How often do you listen to music while you are working? (choice)
  - Always
  - Often
  - Occasionally
  - Rarely
  - Never

- 11b - Which music genre(s) are you listening to concentrate? (multiple choice)
  - Afrobeats
  - Alternative/Indie
  - Ambient
  - Blues
  - Classic Rock
  - Classical
  - Country
  - EDM
  - Film Score
  - Folk
  - Hip-Hop/Rap
  - House
  - Jazz
  - K-Pop
  - Latin/Reggaeton

  - LoFi
  - Metal
  - Pop
  - R&B/Soul
  - Reggae
  - Rock
  - Swing
  - Techno
  - Other

- 11c - Which music genre(s) are you listening to concentrate - Could you specify 'Other' please: (free text - only if 11b include Other)
- 12 - Have you ever played a musical instrument? (choice)
  - Yes
  - Not anymore
  - Never
- 12a - Which Instrument(s)? (multiple choice - only if 12 not Never)
  - Piano or Similar
  - Guitar or Similar
  - Violin or Similar
  - Wind Instruments
  - Brass Instruments
  - Drums or Similar
  - Singing (Voice)
  - Other
- 12b - At what level did you play? (If multiple, select the highest.) (choice - only if 12 not Never)
  - None
  - Beginner
  - Intermediate
  - Advanced
  - Professional
- 13 - Do you think that music influences your concentration? (choice)
  - Yes
  - Maybe
  - No
  - Don't know

Supplementary Text 1.3 Questionnaire Q1B

- 1 - How stressed did you feel during the last phase? (slider)
- 2 - How well were you able to concentrate during the last phase? (slider)
- 3 - How motivated did you feel during the last phase? (slider)
- 4 - Did the last phase feel like it took 3 minutes? (choice)
  - Shorter
  - About right
  - Longer
- 5 - How would you describe your current mood? Please select from the following options. (multiple choice)
  - Happy
  - Sad
  - Anxious
  - Calm
  - Excited
  - Frustrated
  - Relaxed
  - Angry
  - Content
  - Stressed

Supplementary Text 1.4 Questionnaire Q1I

- 1 - How stressed did you feel during the last phase? (slider)
- 2 - How well were you able to concentrate during the last phase? (slider)
- 3 - How motivated did you feel during the last phase? (slider)
- 4 - Did the last phase feel like it took 3 minutes? (choice)
  - Shorter
  - About right
  - Longer
- 5 - How would you describe your current mood? Please select from the following options. (multiple choice)
  - Happy
  - Sad
  - Anxious
  - Calm
  - Excited
  - Frustrated
  - Relaxed
  - Angry

  - Content
  - Stressed

- 6 - Do you know the song? (choice)
  - Yes
  - No
  - Unsure

- 7 - Do you like the song? (slider)

Supplementary Text 1.5 Questionnaire Q2

- 1 - Do you think that music influenced your concentration during the study? (choice)
  - Yes
  - Likely yes
  - Likely not
  - No
  - Don't know

- 2 - Why do you think music did (not) influence your concentration during the study? (free text - only if 1 not Don't know)

- 3 - Feel free to provide any kind of feedback (free text)

Supplementary Text 2 Consent

Informed Consent Document - PIANo-1 Study

Informed Consent Document

for the Study

PIANo-1 Study: Personalized Investigation of Music's Effect on Attention in an N-of-1 trial

[redacted]

Please review the following details before providing your consent.

[redacted] [redacted]

1 | 9

Informed Consent Document - PIANo-1 Study

Introduction

The research is being conducted by [redacted] The team is committed to conducting this study with the highest ethical standards, ensuring your privacy and well-being throughout the process.

Principal Investigator (PI)

- [redacted]

Ethic Committee/IRB

- [redacted]

Your involvement in this research is completely voluntary. If you choose to participate, you will be contributing to important findings that may benefit future studies. Please take your time to review the details carefully, and feel free to reach out if you have any questions or concerns.

Study Information

With the study, we aim to experimentally investigate the individual effect of listening to self-selected music on concentration with an N-of-1 trial. We focus on the outcome measured with a concentration task performed by the study participant and confirm the results with the measured sensor data.

Study Procedures

If you agree to participate, you will be asked to:

During the study periods, you will complete a digital version of the Stroop test. In this task, you will see words that name colors (e.g., "red," "blue") displayed in various ink colors. Your job is to press a specific key to indicate whether the meaning of the word matches the color of the ink (e.g., the word "red" written in red = match; the word "red" written in blue = mismatch).

After each Stroop test session, you will fill out a short questionnaire to rate your self-perceived concentration during the task. During some sessions, you will listen to pre-selected music tracks from various well-known genres. You will choose your preferred music genre in advance, based on your personal taste. During other sessions, no music will be played, allowing comparison between music and no-music conditions. The study will take around 3 minutes per period resulting and a short break in between. We expect 20 to 30 minutes in total.

To participate in this study, you must meet the following conditions:

- You are 18 years or older.
- You are fluent in either German or English.
- You are willing to provide informed consent before the study begins.

Unfortunately, you cannot take part if any of the following apply:

- You are currently enrolled in another intervention study.
- You have colorblindness, as the task requires distinguishing between different ink colors.
- You have hearing loss or hearing related issues like tinnitus.
- You have been diagnosed with epilepsy or any other neurological disorder.
- You are currently experiencing or have a history of substance abuse (e.g., alcohol,
- drugs).

If you are unsure whether any of these apply to you, please feel free to ask the study team for clarification.

Study Risks and Benefits

This study involves minimal risk. However, some participants may experience the following:

- Mild mental fatigue or frustration during the Stroop test, as it requires sustained concentration and quick responses.
- Temporary stress or performance pressure, especially during timed tasks.
- Auditory discomfort, particularly if you are sensitive to certain types of music or sounds, even at normal listening volumes.
- Emotional stress can occur, if the song contains explicit language.

You are free to pause or withdraw at any time if you feel unwell or uncomfortable. While there may be no direct personal benefit to you, participating in this study may offer the following:

- An opportunity to learn more about how your concentration may be affected by music.
- You will be contributing to scientific research that aims to better understand how music influences cognitive performance, which could help improve future educational or therapeutic practices.

Please note that participation is voluntary and should not be expected to result in medical or psychological treatment.

Participant Rights and Data Handling

Confidentiality

Participants' information will be stored securely at [redacted] Only the study team and trained researchers will have access to the raw data. After the data is processed, anonymous data will be shared.

Data Collection and Storage

The study will be conducted with a python implemented tool. For that, you will be assigned to a randomly generated ID to match the answers and sensor data afterwards. A list containing the participants' name and the randomly generated pseudonym will not be created, hence the data will be stored anonymously. During data collection, the presence of the study participant allows the data to be linked to them. The sensor data will be streamed locally on the machine over the lab streaming layer to collect data from different sensors. For that, a local network will be generated protected from outside access to connect the sensors to the recorder tracking the measurements from the different devices. Questionnaires as well as sensor data will be stored with the randomly generated pseudonym and without any participants' name or contact information. While collecting the data, the data will be stored on a HPI owned laptop. Directly after the participant conducted the trial, the data will be encrypted to ensure that no third party can access the raw data. The encrypted data will be stored anonymized on HPI infrastructure such as Nextcloud and the scientific compute cluster for processing and analysis with restricted access.

Data Sharing

After the study is analyzed, the data will be published. However, as we expect a small number of participants, it might be the case that participants can be identified by age and gender combination. If there is only one participant with a certain combination, the baseline characteristics of the participants will not be published with the data set and only aggregated information will be shared in the publication (like how many people are female, how many people are between 30-40 years old). With that, we ensure that even if the data is published, the privacy of individuals is well protected.

Study Withdrawal

The participant has the right to withdraw from the study. Due to anonymization, the participant can only withdraw during the onsite meeting. Afterwards it would not be possible to identify the participants data due to anonymization.

Acknowledgement

If you have any questions about this study, please contact the responsible PI or research team. If you have concerns about your rights as a research participant, you may contact the ethics board. All contact information is provided at the beginning of this document.

Contact details

Principal Investigator

- [redacted]

Participating Researcher

- [redacted]

Information on data protection

We process your personal data in compliance with the applicable data protection regulations, in particular the EU General Data Protection Regulation (GDPR) and the Brandenburg Data Protection Act (BbgDSG).

The data controller is:

[redacted]

[redacted]

Purpose of the processing

The data is collected as part of the PIANo-1 study and processed for analysis. The aim is to gain insights into the individual influence of music on concentration and to further develop and validate methodologies for analysing N-of-1 studies.

Lawfulness of processing

The legal basis for data processing is your consent to the processing of personal data (Art. 6 para. 1 sentence 1 lit. in conjunction with Art. 9 para. 2 lit. a GDPR).

Type of data

The following data categories are processed as part of the study:

- **Sociodemographic information** (age, gender): personal data.
- **Questionnaire data** (music preferences, listening habits, self-assessments, language): personal data.
- **Study data** (task performance in the Stroop test: response accuracy, reaction times, session and block identifiers): pseudonymized research data.
- **Sensor data** (EEG, heart rate/heart rate variability using PPG, electrodermal activity, skin temperature, motion sensors): special categories of personal data within the meaning of Art. 9 GDPR (health and physiological data).

All data is collected and stored in pseudonymized form. Identification can only be carried out by means of comparison by the study participants. Once data collection has been completed, the data is permanently anonymized for scientific evaluation.

Recipients of your data

Your data will not be transmitted to third parties or other recipients within the [redacted]

Duration of data processing

The data is anonymized immediately after collection and once it has left the test environment. The anonymized raw data is deleted after 36 months at the latest. Processed anonymized data is published with the analysis.

Your rights:

You have the right to obtain from the controller confirmation as to whether or not your personal data is being processed, and, if that is the case, access to the personal data and additional information (right of access). You also have the right to obtain from the controller without undue delay the rectification of inaccurate personal data. If the legal requirements of Art. 17 or 18 GDPR are met, you are entitled to the erasure of your personal data or to a restriction of processing. Please note that restricted processing of the data may not be possible in every instance. You have the right to receive your personal data in a structured, standard and machine-readable format or to request the transfer to another controller (right to data portability, Art. 20 GDPR). Furthermore, you may object to the processing of your personal data under the conditions set forth in Art. 21 GDPR.

In order to exercise your rights, we kindly request that you contact:

[redacted]

Access to your personal data can be requested from the Chief Information Officer [redacted]

[redacted]

You can contact the [redacted]

[redacted]

[redacted]

[redacted] [redacted]

If you think that the processing of your personal data infringes the GDPR, then you have the right to lodge a complaint with the supervisory authority for data protection.

Consent

You will be given a copy of this form to keep. **Participation in Research is voluntary.** You have the right to say "No" to this study now or at any point without penalty. If you wish to take part in this study, please sign below.

☐ I consent that I have read and understood the consent and study information provided. I agree that the collected anonymized data can be published as described above.

☐ I hereby consent to the processing of personal data associated with participation in the study, including health data in the form of exclusion criteria and sensor data (EEG, EDA, and PPG).

______________________ ____

Date Participant's Signature for Consent

______________________ ____

Date Person Obtaining Consent
